# Allergic Reactions to Serine Protease-Like Proteins of *Staphylococcus aureus*

**DOI:** 10.3389/fimmu.2021.651060

**Published:** 2021-03-23

**Authors:** Maria Nordengrün, Goran Abdurrahman, Janina Treffon, Hannah Wächter, Barbara C. Kahl, Barbara M. Bröker

**Affiliations:** ^1^Department of Immunology, Institute for Immunology and Transfusion Medicine, University Medicine Greifswald, Greifswald, Germany; ^2^Institute of Medical Microbiology, University Hospital Münster, Münster, Germany; ^3^Institute of Hygiene, University Hospital Münster, Münster, Germany

**Keywords:** cystic fibrosis, *Staphylococcus aureus*, allergy, type 2 immune response, IgE, Th2 cells

## Abstract

In cystic fibrosis (CF) infectious and allergic airway inflammation cause pulmonary exacerbations that destroy the lungs. *Staphylococcus aureus* is a common long-term colonizer and cause of recurrent airway infections in CF. The pathogen is also associated with respiratory allergy; especially the staphylococcal serine protease-like proteins (Spls) can induce type 2 immune responses in humans and mice. We measured the serum IgE levels specific to 7 proteases of *S. aureus* by ELISA, targeting 5 Spls (76 CF patients and 46 controls) and the staphopains A and B (16 CF patients and 46 controls). Then we compared cytokine release and phenotype of T cells that had been stimulated with Spls between 5 CF patients and 5 controls. CF patients had strongly increased serum IgE binding to all Spls but not to the staphopains. Compared to healthy controls, their Spl-stimulated T cells released more type 2 cytokines (IL-4, IL-5, IL-13) and more IL-6 with no difference in the secretion of type 1- or type 3 cytokines (IFNγ, IL-17A, IL-17F). IL-10 production was low in CF T cells. The phenotype of the Spl-exposed T cells shifted towards a Th2 or Th17 profile in CF but to a Th1 profile in controls. Sensitization to *S. aureus* Spls is common in CF. This discovery could explain episodes of allergic inflammation of hitherto unknown causation in CF and extend the diagnostic and therapeutic portfolio.

## Introduction

Cystic fibrosis (CF) is the most common life-shortening genetic disorder, afflicting around 7/100 000 of the general population in the US and the European Union (1). Recurrent bacterial lung infection and persistent airway inflammation gradually destroy the lung, ultimately resulting in respiratory failure (2). The causative agents are bacteria, prominently *Staphylococcus aureus* (*S. aureus*) and *Pseudomonas aeruginosa*, but also fungi, especially the ubiquitous *Aspergillus fumigatus* (*A. fumigatus*) (3). Persistent colonization with *S. aureus* occurs early in the disease course in up to 70% of CF patients, a much higher percentage than in the general population (2, 4). In the long term, the pathogen adapts to the host, gradually reducing its virulence during airway infection (5). Nevertheless, recurrent pulmonary exacerbations gradually worsen the lung function and clinical condition of CF patients (6). Prevention and therapy of chronic bacterial and fungal inflammation are therefore key in the treatment regimen of CF (6–8).

Besides infections, allergic immune responses play a crucial role in disease progression of CF. These are frequently associated with sensitization to *A. fumigatus* with 1-15% of patients suffering from allergic bronchopulmonary aspergillosis (ABPA), accelerating the decline of respiratory function (8, 9).

*S. aureus* is a frequent colonizer of nose and skin in the general population, but given appropriate circumstances, the microorganism can turn into a dangerous pathogen and cause a broad range of infections (10). *S. aureus* is also associated with allergic airway inflammation (11, 12). Recently we showed that the staphylococcal serine protease-like proteins (SplA – SplF) elicit a type 2-biased immune response in healthy individuals and especially in asthma patients. We observed serum IgE binding to these bacterial proteins in most asthmatics and a minority of healthy individuals. SplD was able to induce allergic airway inflammation *de novo* when applied intratracheally in a murine allergy model (13, 14).

To elucidate whether CF patients – many of whom are persistently exposed to *S. aureus* in their airways – react with type 2 inflammation to the Spls, we examined their specific IgE and T cell responses.

## Materials and Methods

### Blood Donors

Serum samples from CF patients (n = 76) were obtained at the Institute of Medical Microbiology, University Hospital Münster, Germany. They comprise two cohorts, a multicenter study (n = 62) (5, 15) as well as a two center study that was conducted in Münster (n = 14). Samples from healthy individuals were obtained from in-house volunteers (n = 46). The median age of CF patients was 14.9, 52 patients (68.5%) were male, 24 (31.5%) were female. In 44 subjects from the multicenter study the *S. aureus* nasal colonization status was known; 28 were *S. aureus* nasal carriers and 16 were non-carriers. The median age of the healthy subjects was 23, 13 (28.2%) were male and 33 (71.8%) female; 16 (34.8%) were persistent *S. aureus* carriers. Five CF patients (from the two center study) and five healthy volunteers additionally donated peripheral EDTA blood samples. All blood donors gave informed consent (Approvals of the responsible Ethics Committees; Greifswald: IIIUV 23/06a, BB007/17; Münster: 2007-496-f-S, 2014-054-f-S).

### Antigens

Recombinant Spls were generated as described (14). Lyophilized staphopain A and B were purchased from Sigma-Aldrich and reconstituted in PBS. When used in cell culture assays, the proteins were denatured at 95°C for 30 min.

### Antibody Response

Enzyme-linked immunosorbent assays (ELISAs) were performed as previously described (14). Briefly, wells of 96-well microtiter plates (MaxiSorp, Nunc) were coated with 5 μg/mL recombinant Spls (50 μL/well). Serum samples were diluted 1:5 and added in duplicate wells. The bound IgE-antibodies were detected with biotinylated rabbit anti-human IgE antibody (10 µg/mL; antibodies online) followed by Streptavidin-HRPO (1:333; Dianova). TMB substrate reagent was added for 10 min, and the reaction was stopped with 20 µl 2N sulfuric acid. The optical densities (OD) were measured at 450 nm in Infinite M200 Pro (Tecan Austria GmbH). Negative controls were processed without the addition of serum. Each assay was repeated on two separate days.

### Cellular Response to the Spls

PBMCs were isolated from 30 mL of whole blood using standard gradient methods and cryopreserved until analysis. After thawing, CD14+ monocytes were isolated from PBMCs by positive selection using CD14 MicroBeads (Miltenyi Biotec 30-050-201). Untouched T cells were isolated from the CD14-negative fraction using PAN T cell isolation kits (Miltenyi Biotec 130-096-535). The purity of the isolated T cells was assessed by flow cytometry and was > 95%.

The purified T cells were co-incubated with irradiated CD14+ feeder cells at a ratio of 10:1 in RPMI medium (PAN Biotech, P04-17500) supplemented with 5% human serum (PAN Biotech, P30-2401), 100 IU/mL penicillin, 200 µg/ml streptomycin, 4 mM glutamine, 50 µM β-mercaptoethanol, 1.0 mM sodium pyruvate, 0.1 mM non-essential amino acids (Sigma, M7145-100M). Cells were seeded in 24-well flat bottom plates and stimulated with a cocktail of recombinant SplA, SplB, SplD, SplE, and SplF (5 mg/mL each). On day 5, 750 µL of the medium was replaced by fresh medium supplemented with 20 IU/mL human recombinant IL-2 (Miltenyi Biotec).

On day 9, the supernatant was taken and stored at -80°C until analysis. The cytokine concentrations in the supernatant were measured using a 13-plex cytometric bead array (LEGENDplex Human Inflammation Panel, BioLegend 740721), and cytokine concentrations were determined with the corresponding LEGENDplex software.

The T cells were harvested in PBS and stained using fluorochrome-conjugated antibodies (**Supplementary Table 1A**). NIR (Biolegend, 423106) was used to exclude dead cells. Data were acquired on an LSR II (BD Bioscience, San Jose, CA, USA) and FlowJo (Treestar, Ashland, OR, USA) software was used for analysis. FSC-A vs. FSC-H blots identified singlets. After gating on live T cells (NIR-CD3+), CD4+ Th cell subsets were identified by their chemokine receptor expression patterns as shown in (**Supplementary Table 1B**).

## Results

### Increased Spl-Specific Serum IgE Levels in CF Patients

**Figure 1:**
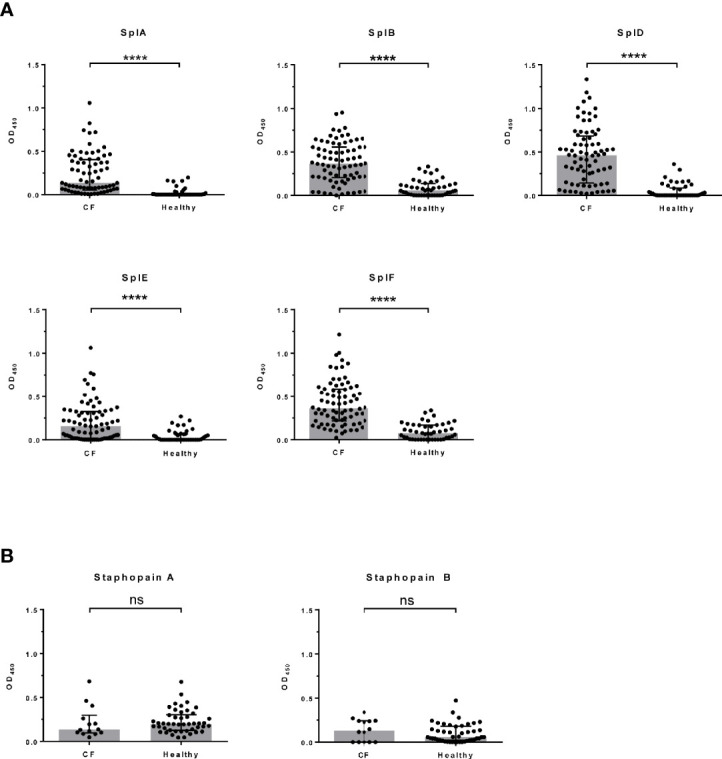
*S. aureus* protease-specific IgE in sera of CF patients and healthy adults. Specific serum antibody binding was determined by ELISA. Each data point represents the mean of two technical replicates. Spl-specific serum IgE levels were significantly higher in CF patients (n = 76) than in healthy controls (n = 46) **(A)**, whereas no significant differences were seen in the staphopain A and staphopain B specific IgE levels (CF: n = 14; controls: n = 46) **(B)**. Medians (grey bars) with interquartile ranges are shown. ****P < 0.0001; Mann Whitney U test. CF, cystic fibrosis, ns, not significant, OD, optical density.

We analyzed 76 CF patients from two cohorts, a multi-center study (n = 62) (5, 15) and a two-center study (n = 14), as well as 46 healthy adults. All patients were persistently colonized and recurrently infected with *S. aureus* in their airways. We quantified Spl-specific IgE in the sera by ELISA. The antigens SplA, SplB, SplD, SplE, and SplF were tested, and we found IgE binding to all of them to be strongly elevated in CF patients compared with controls (**Figure 1A**). Only a minority of the healthy adults had measurable concentrations of Spl-specific serum IgE. The difference was robust and remained highly significant when the two patient cohorts were tested separately (see **Supplementary Figure 1**). There was pronounced variability in the patterns of each patient’s IgE binding to the five Spls. This likely reflects the patients’ history of exposure to these enzymes: Spl-specific IgE tended to be higher in CF patients that were persistently colonized with *S. aureus* not only in the lung but also in the nose; in the case of SplA this difference reached significance (**Table 1**). While all Spls are encoded in one operon that is present in around 80% of clinical *S. aureus* isolates, the composition of this operon is variable, indicating that in *S. aureus*-infected CF patients the immune system is confronted with different subsets of the Spl proteins (16).

**Table 1:** Correlation between nasal *S. aureus* carrier status and anti-Spl IgE.

Spl	Nasal *S. aureus* carriers^a^ (n = 28)		*S. aureus* non-carriers (nose) (n = 16)		*P*-value^b^
	Mean ± SD	Median	Mean ± SD	Median	
SplA	0.29 ± 0.28	0.23	0.12 ± 0.18	0.05	0.035*
SplB	0.41 ± 0.26	0.36	0.26 ± 0.25	0.19	0.069
SplD	0.40 ± 0.29	0.38	0.52 ± 0.42	0.47	0.479
SplE	0.21 ± 0.25	0.13	0.11 ± 0.17	0.03	0.051
SplF	0.47 ± 0.29	0.43	0.31 ± 0.19	0.27	0.062

a ) Information about nasal S. aureus colonization was available for 44 CF patients, 28 carriers and 16 non-carriers.

b ) Mann-Whitney U test; *) P < 0.05.

Since protease activity is common in allergens, we next analyzed the IgE response against two other cysteine proteases of *S. aureus*, staphopain A (ScpA) and staphopain B (SspB). In contrast to the remarkably increased IgE response to the Spls in CF, the staphopain-specific IgE serum levels did not differ between CF patients (n = 14; subjects of the two-center study) and healthy controls (n = 46) (**Figure 1B**). This highlights that the ability to induce a strong IgE response is a specific property of the Spls rather than a general feature of *S. aureus* proteases or, in fact, staphylococcal antigens in general and corroborates earlier findings that *S. aureus* antigens can elicit immune responses of different quality in the same individual (14, 17). It is plausible to assume that the Spls’ proteolytic activity has a role in causing the type 2 bias of the specific antibody response. The Spls of *S. aureus* are known to have distinctive and very selective preferences for cleavage motifs, indicating a narrow substrate range (18, 19). However, the knowledge about the Spls’ pathophysiological substrates is very limited (20, 21). The extensive and long-term exposure of the CF patients’ airways to *S. aureus* drives a strong antibody response to many *S. aureus* antigens, documented by high specific IgG titers (5, 15). However, this pronounced humoral immune reaction to the bacteria cannot be the only reason for the sensitization to the Spls in CF, which is very selective.

### Th2 Bias in Spl-Reactive T Cells of CF Patients

Immunoglobulin class switch to IgE requires the help of antigen-specific Th2 cells. Therefore, we studied the Spl-specific T cell memory response in CF patients (n = 5) and healthy controls (n = 5) and compared cytokine production and phenotype of the Spl-stimulated T cells. We isolated and co-cultured T cells and CD14^+^ antigen-presenting cells from peripheral blood, stimulated them with a mixture of recombinant SplA, SplB, SplD, SplE, and SplF (each at 5 µg/mL) for nine days and then measured cytokines in the cell culture supernatants.

**Figure 2:**
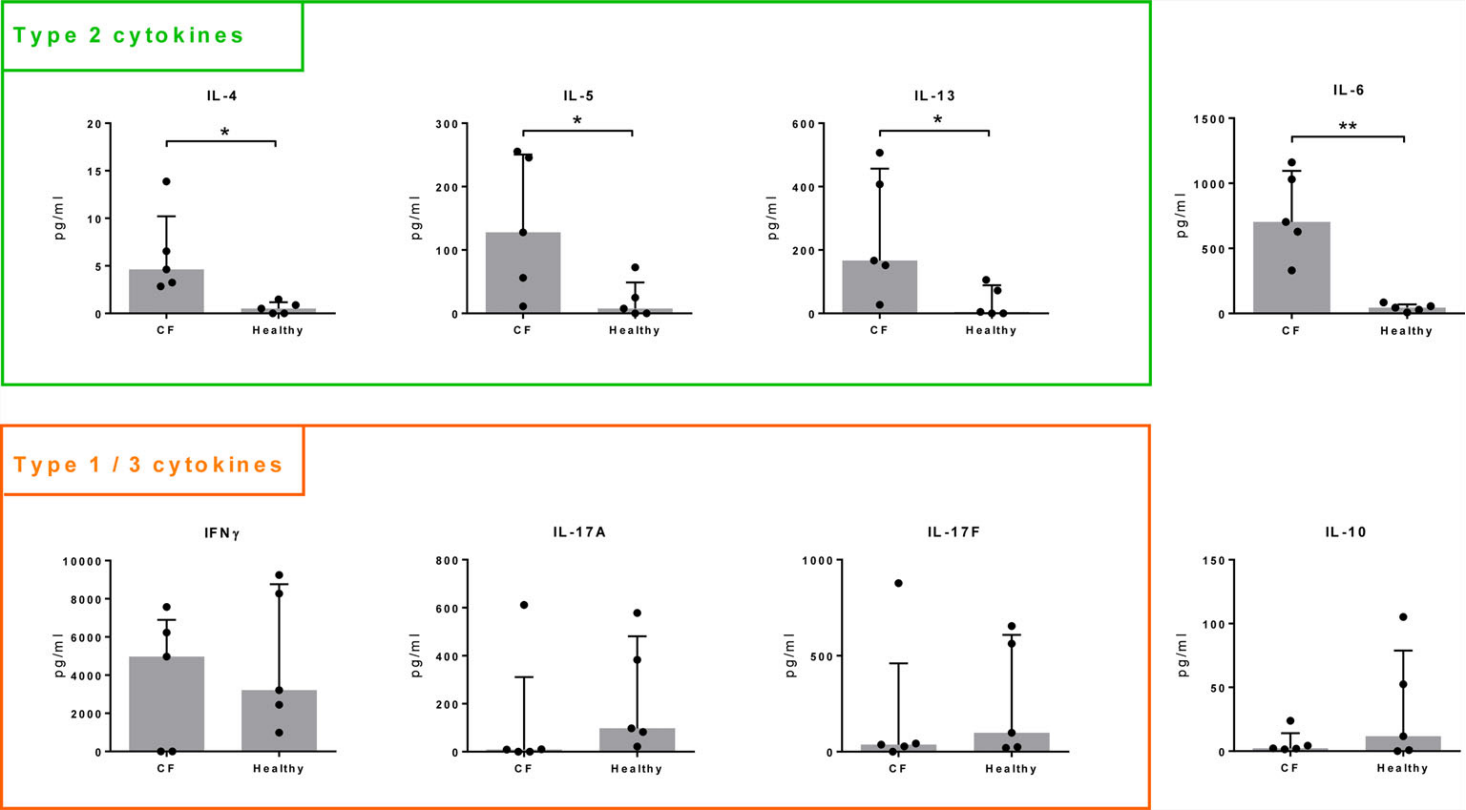
Cytokine production by Spl-stimulated T cells. T cells were isolated from whole blood of CF patients (n = 5) and healthy volunteers (n = 5) and stimulated with a mixture of recombinant SplA, SplB, SplD, SplE and SplF (5 µg/mL each) in the presence of CD14+ antigen-presenting cells. Supernatants were taken at day 9 and cytokine concentrations measured by a cytometric bead array. Concentrations were normalized to 1 million T cells. Production of Th2 cytokines (IL 4, IL 5, IL 13) and IL 6 was significantly higher in CF patients compared to healthy controls, IL 10 release in tendency lower, while there were no significant differences in the concentrations of IFNγ, IL 17A, or IL 17F. Medians (grey bars) with interquartile ranges are indicated; *P < 0.05; **P < 0.01; Mann Whitney U test. CF, cystic fibrosis.

In healthy controls, type 2 cytokines (IL-4, IL-5, IL-13) were of low concentration or below the threshold of detection. In comparison, release of these cytokines was significantly increased in all cultures from CF patients. Similarly, IL-6 production was significantly higher in T cells isolated from CF patients than in those from controls, whereas IL-10 release tended to be lower in CF (**Figure 2**). We did not observe significant differences for IFNγ, IL-17A, IL-17F (**Figure 2**) nor for TNF, IL-9, IL-21 or IL-22 (not shown).

**Figure 3:**
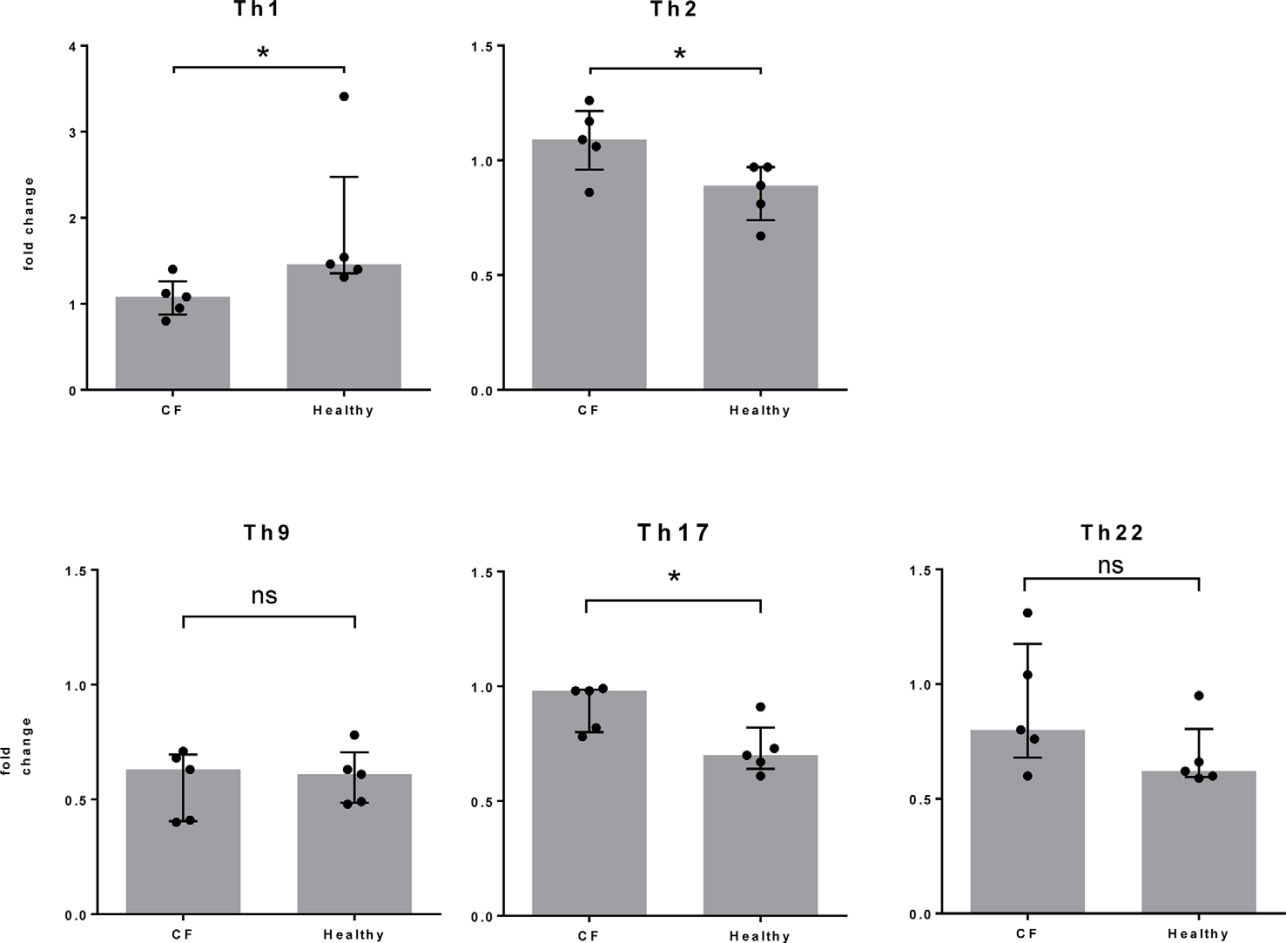
T cell differentiation following Spl stimulation. From the same cell cultures described in **Figure 2**, T cells were harvested at day 9 and the proportion of each T cell subtype was determined by FACS. Fold changes between unstimulated and Spl-stimulated cells are shown. Compared to healthy controls, the changes in Th2 and Th17 cells were significantly higher and those in Th1 cells significantly lower in Spl-stimulated T cells from CF patients. *P < 0.05; Mann Whitney U test. CF, cystic fibrosis.

At the same time point, nine days after Spl stimulation, we assessed the phenotype of the T cells by flow cytometry and determined the proportions of CD4^+^ T cell subtypes according to their chemokine receptor expression. Focusing on changes in the T cell subtype composition due to Spl exposure, we found a stronger Th2 and Th17 cell response in CF patients, whereas Th1 cells dominated the reaction in the control individuals (**Figure 3**). The slightly increased percentage of Th17 cells in CF patients was not reflected in the release of IL-17 in cell culture. This is not easily explained. It may reflect the known plasticity of Th17 cells, which developed differently in CF patients than in controls (22, 23).

## Discussion

The sensitization of CF patients to antigens of *S. aureus* that is colonizing and infecting their airways is reminiscent of ABPA, where a type 2 airway inflammation specific to the ubiquitous fungus *A. fumigatus* destroys lung function if left untreated (8, 24). In some patients the Spl-directed IgE response was very strong, and we propose that this could be an unrecognized cause of allergic lung exacerbation in CF patients harboring *S. aureus* in their airways. Type 2 immune responses may also favor bacterial colonization and infection because they counteract the immune clearance mechanisms, which are of a type1/type 3 profile. However, in our study, anti-Spl IgE levels did not differ significantly between CF patients who experienced lung exacerbations during the study period and those who did not, nor did they correlate with lung function (FEV_1_% predicted). Probably our CF cohort was too small and too heterogeneous to show the influence of a single factor on the complex pathogenesis. Even in ABPA a bronchial provocation test was required to reveal the eosinophilic inflammation and reduction of FEV_1_ in CF patients that were sensitized to *A. fumigatus* (25). Moreover, our analysis of the T cell response to Spls is limited by the small numbers of tested persons. The T cell analyses required substantial amounts of fresh blood, which only 5 patients in the second study cohort could safely provide. Nevertheless, the results clearly demonstrate skewing of the Spl-specific memory towards a type 2 profile in CF, possibly accompanied by a loss of tolerance that is indicated by the reduced IL-10 production. These findings corroborate the results of our IgE and cytokine measurements, underlining the specific type 2 quality of the adaptive immune response to the Spls of *S. aureus* in CF.

The discovery of allergic reactions to the Spls of *S. aureus* opens a new avenue for research and therapy. Further studies are now warranted to find out if CF patients develop allergic reactions to other colonizing or infecting bacteria as well. The quest for bacterial allergens is still in its beginning (21). However, sensitization to staphylococcal enterotoxins (SE) is well documented in chronic rhinosinusitis with nasal polyps where it is an independent risk factor for co-morbid asthma (26). Besides SE-specific IgE, many asthmatics have elevated serum IgE against *S. aureus* Spls (14). It is possible that sensitization to persistent colonizing and infecting bacteria significantly contributes to disease progression in some CF patients. In this case the therapeutic portfolio may be extended, because agents that selectively interfere with type 2 inflammation without hampering anti-microbial defense mechanisms are rapidly becoming available.

## Data Availability Statement

The original contributions presented in the study are included in the article/**Supplementary Material**. Further inquiries can be directed to the corresponding author.

## Ethics Statement

The studies involving human participants were reviewed and approved by Approvals of the responsible Ethics Committees; Greifswald: IIIUV 23/06a, BB007/17; Münster: 2007 496-f S, 2014-054 f S. The patients/participants provided their written informed consent to participate in this study.

## Author Contributions

Study concept and design: BB and BK. Designed and performed experiments: MN, GA, and JT. Wrote the manuscript: GA, BB, MN, BK, and JT. Analyzed the data: GA, BB, MN, BK, and HW. All authors contributed to the article and approved the submitted version.

## Funding

This study was funded by grants from: The German Research Foundation (DFG) [CRC TRR34 to BK and BB], The German Research Foundation [GRK1870 to GA and MN], European Social Fund [ESF/14 BMA55-0037/16 “Card-ii-Omics” to MN and BB], and Mukoviszidose e.V. [S05/07 to BK]. We acknowledge support for the Article Processing Charge from the DFG (German Research Foundation, 393148499) and the Open Access Publication Fund of the University of Greifswald.

## Conflict of Interest

The authors declare that the research was conducted in the absence of any commercial or financial relationships that could be construed as a potential conflict of interest.
