# Supplementary material for: Allergic Reactions to Serine Protease-Like Proteins of Staphylococcus aureus
Source: Front Immunol. 2021 Mar 23;12:651060. doi: 10.3389/fimmu.2021.651060 (PMC8021911; doi:10.3389/fimmu.2021.651060)
Supplement: Supplementary file 1 [file DataSheet_1.pdf]

A

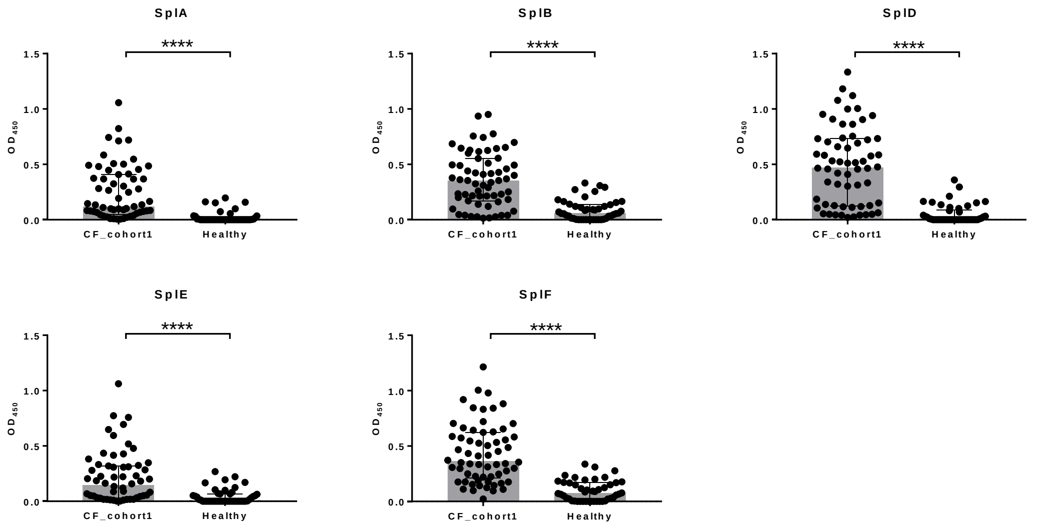
SplA
SplB
SplD
SplE
SplF
****
OD450
CF_cohort1
Healthy
1.5
1.0
0.5
0.0

B

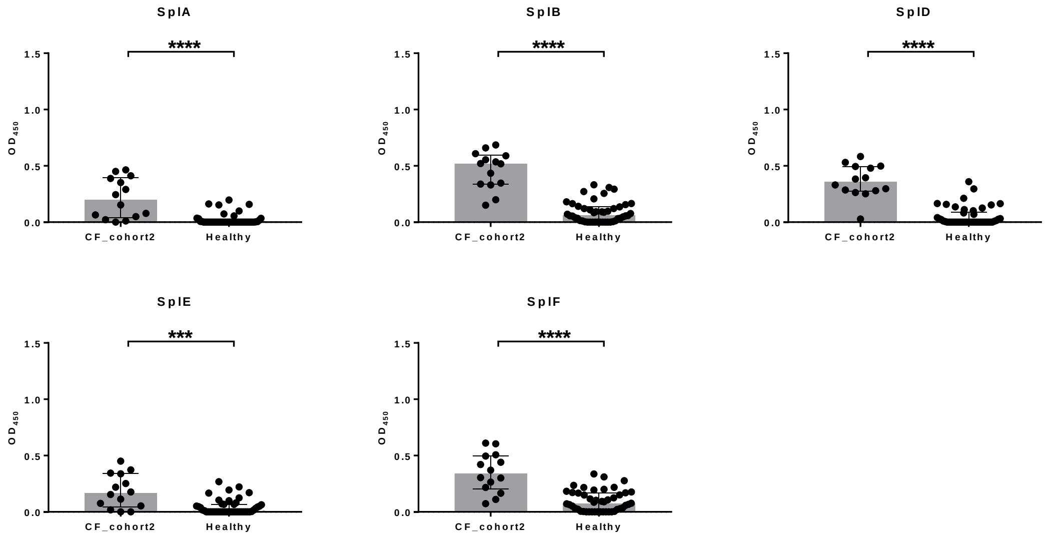
SplA
SplB
SplD
SplE
SplF
****
***
OD450
CF_cohort2
Healthy
1.5
1.0
0.5
0.0

C

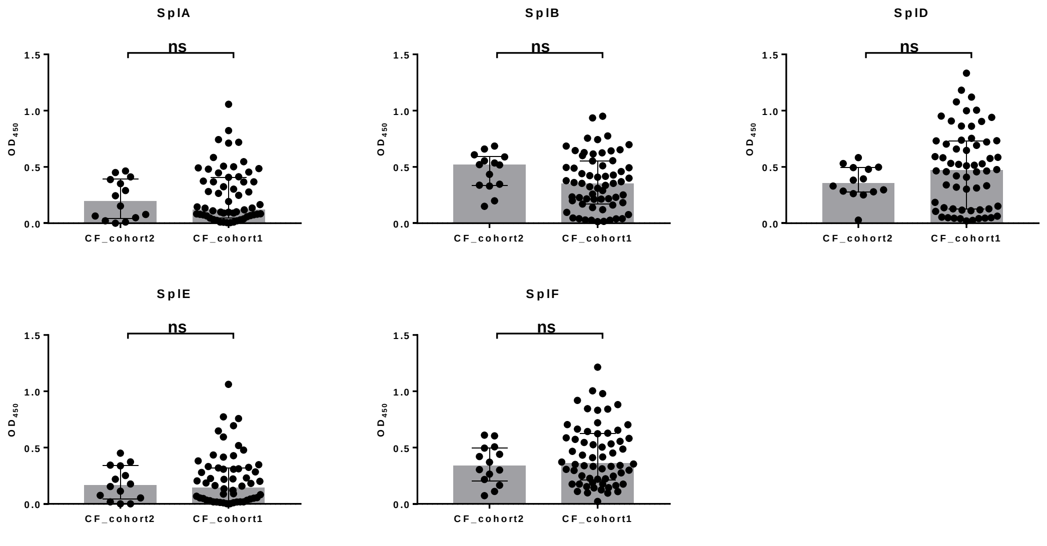
SplA
SplB
SplD
SplE
SplF
ns
OD450
CF_cohort2
CF_cohort1
1.5
1.0
0.5
0.0

Supplementary Figure 1. *S. aureus* protease specific IgE in sera of CF patients and healthy adults Specific serum antibody binding was determined by ELISA. Each data point represents the mean of two technical replicates. Spl-specific serum IgE levels were significantly higher in the two CF patients cohorts (cohort1: n=62, cohort2: n=14) when they were independently compared to healthy controls (n = 46) (A & B). No significant differences were seen in the specific IgE levels when the two CF cohorts were compared to each other; both cohorts had equally strong increased IgE levels (C). Medians (grey bars) with interquartile ranges are shown. *** $P < 0.001$; ****$P < 0.0001$; Mann Whitney U test. CF: cystic fibrosis, ns: not significant, OD: optical density.

Supplementary Table 1A: Antibodies

**Antigen**	**Order number**	**Dye**	**Channel**	**Manufacturer**	**Clone**	**Host**	**Isotyp**
CD3	130-114-710	VioBlue	V450	Miltenyi	REA613	human	IgG1
CD4	130-113-792	VioGreen	V525	Miltenyi	REA623	human	IgG1
CD183 (CXCR3)	130-118-673	VioBright-FITC	B525	Miltenyi	REA232	human	IgG1
CD194 (CCR4)	130-117-525	APC	R670	Miltenyi	REA279	human	IgG1
CD196 (CCR6)	130-100-382	PEVio770	YG780	Miltenyi	REA190	human	IgG1
anti-CCR10	130-104-868	PE	YG582	Miltenyi	REA326	human	IgG1
NIR	423105		R780	Biolegend			

Supplementary Table 1B: T cell subsets

**Subset**	**Markers**
Th1	CD4+CXCR3+
Th2	CD4+CCR4+CCR6-
Th9	CD4+CCR4–CCR6+
Th17	CD4+CCR4+CCR6+
Th22	CD4+CCR4+CCR6+CCR10+
